# Supplementary material for: One size fits all? Transferring social mindfulness measures to HRI
Source: PLoS One. 2026 Jan 2;21(1):e0339959. doi: 10.1371/journal.pone.0339959 (PMC12758777; doi:10.1371/journal.pone.0339959)
Supplement: Attachment — Submitted filename: Response_to_Reviewers.docx [file pone.0339959.s002.docx]

# Rebuttal letter

Dear Reviewers,

Thank you both for your comments and for taking the time and effort to provide such in depth feedback. Your comments are insightful and stimulated an academic discussion among the research team that in our opinion further improved the paper. Below we respond to each of the points you raised separately and explain how and where we addressed them in the revised version of the manuscript (the revised version entails the track changes and is also the version that the line numbers refer to):

## 5. Review Comments to the Author Reviewer 1:

Reviewer #1: 1. *The abstract effectively identifies a methodological gap but provides little detail on how the two studies were structured or what types of robots and interaction settings were used. Clarifying the experimental design and the scope of interaction contexts would help readers better understand the validity and generalizability of the findings.*

- We added the requested information and agree that providing more information on robots used, experimental setup and interaction context in the abstract makes it easier for others to understand the meaning, context and generalizability of our research.

*2. While the paper challenges the direct transfer of HHI measures to HRI, the abstract lacks a clear articulation of what new insights or frameworks emerge from this work. The authors should elaborate on how their findings advance the theoretical understanding of social mindfulness in HRI or guide future methodological development in robot interaction research.*

- We added a few sentences on theory on social mindfulness in robots and anthropomorphism as a dynamic perceptual concept. While our findings have theory implication, we believe that the main contribution of our research is methodological. Our outcomes show how sensitive research measures are to context and experimental design. Hence, we also highlighted that future research should engage in cautious reflection when transferring measures from HHI to HRI, in our abstract.
- Based on your remark we also added a few sentences about theory contributions and methodological development to the discussion/future research section of the paper.

*3. The introduction should clearly conclude with a distinct paragraph that highlights the novel contributions of your work.*

- Below the introduction of both study one and study two (line 194 and 227) we added two paragraphs that highlight the novel contribution of each respective study to the field.

*4. The literature review should benefit from more explorations of previous studies.*

- We have included references throughout the text, merging results from HHI and HRI in terms of SoMi to highlight better the research gap our manuscript aims to elucidate. We also want to stress that we used Vancouver referencing style as requested by PlosOne, which means references are only included as numbers in the manuscript and might therefore easily be overlooked. We also reformulated our discussion of earlier research in some parts to elaborate more on previous research findings (e.g. line 164 or 430 onwards). However, in the case that you know of additional relevant literature we would be thankful for any recommendation.

*5. The discussion section needs to be expanded to more thoroughly analyze the results.*

- Thank you for this remark. We agree that a more thorough interpretation of the results in the discussion section would also make the connection to theory, methods and previous research more obvious for the reader. We added multiple sentences on the interpretation of our result to the discussion (see lines: 640-648,672-681, 687)

*6. The first paragraph of the conclusion should succinctly summarize the contributions of the study in past tense.*

- The manuscript which you reviewed had no conclusion paragraph, but we added one at the end of the manuscript and stuck to the past tense when it was grammatically right to do so.

*7. The second paragraph of the conclusion should provide clear and actionable future recommendations.*

- We added concrete recommendations for future research to multiple sections in our discussion (see lines: 644, 680, 706-713, 728, 749)

*8. Some equations are not properly cited.*

- We looked through the manuscript and could figure out which equations you were referring to. Would you be so kind and tells us the line in which you found them, so that we can adjust the missing reference?

*9. Sections and subsections are not numbered, and this confuses the division of the manuscript.*

- Thank you for pointing this out, we added numbers to all section to make the division of the manuscript clearer.

Kind regards,

The research team

## Reviewer 2

*Reviewer #2: This article provides a clear and critical assessment of how the Social Mindfulness Paradigm (SoMi), originally developed to measure prosocial behavior between humans, applies to human-robot interaction (HRI). The key finding is that the traditional SoMi task may not validly capture prosociality in HRI. The authors highlight the need for methodological adjustments when transferring human-based psychological tools to robots, especially regarding stimulus relevance, ecological validity, and anthropomorphism.
The study addresses an interesting and emerging research area and presents a thorough investigation. However, a few questions arise:*

*1. If the authors recognized that a three-day interaction might be too short for prosocial behavior to develop, why wasn’t the duration extended?*

*Dear Reviewer 2, there are two reasons for not extending the duration:*

- Reason 1: Preregistration and research gap: At the time when study 1 was conducted, there was not much literature on long time interactions with robots and their effect on pro-social behavior. Therefore, we designed and pre-registered the experiment with the belief that three-days were enough to have a meaningful experimental manipulation. When looking at measures regarding perceived anthropomorphism we also see that the manipulation worked. We have additional measures on perceived warmth and competence also indicating chages, which we did not include in the paper as they are outside the scope of the current manuscript. Further, at the time we did not yet know the outcomes of our manipulation as we pre-registered the experiment so that the data would be analyzed after they were collected, to not confound our data collection by trying to confirm our hypothesis.
- Reason 2: Time and money restrictions. The data was collected by 3 research master students one of whom is author on this paper. Their study trajectory did not allow for another time window. Further, the money to compensate the participants was not enough to ask for more time investments from the participants' side.

However, we agree with you that an extended period of interaction would have been interesting to explore and in hindsight we would have wished for more funding and the means to conduct such research. Hence, added this as something for future studies to explore and explained our constraints (line 727-732). Still, we believe that study 1 was insightful. It showed that in contrast to our expectations social mindfulness as a behavioral measure (at least with the set of items included in study 1) does not change as fast as perceptual measures like anthropomorphism. This is something to build on in future research that wants to investigate changes over time.

*2. In Study 1, what was the rationale for limiting daily interaction with Cozmo to at least 20 minutes rather than allowing longer sessions?*

- We think there is a misunderstanding of our wording here. This is a lower limit we write about. It means the participants had to interact with Cozmo for a minimum of 20 minutes. We see that reader might get confused and therefore added a more precise description to the manuscript (line 324-326):

“Participants were instructed to interact with Cozmo for at least 20 minutes each day for three consecutive days. We only limited the minimum interaction time per day, but participants were allowed to spend more time interacting with Cosmo with no upper interaction limit. We also controlled for the minimum interaction times with the Cosmo app.”

Kind regards,

The research team
