# Supplementary material for: One size fits all? Transferring social mindfulness measures to HRI
Source: PLoS One. 2026 Jan 2;21(1):e0339959. doi: 10.1371/journal.pone.0339959 (PMC12758777; doi:10.1371/journal.pone.0339959)
Supplement: Attachment — Submitted filename: Response_to_Reviewers_2.docx [file pone.0339959.s003.docx]

# Rebuttal letter

Dear Reviewers,

Thank you for reading our manuscript again and providing your feedback. Your feedback has improved the manuscript, and we want to thank you for that. We are very happy that you deem many of your comments addressed adequately in our revised version of the manuscript. Let us react to the remaining ones.

**Comments and reactions:**

*Reviewer #1: Well responded, however, the abstract still lack more info from the results, it is overly lengthy on general information.*

Dear Reviewer 1,

We tried to address your previous concerns regarding the abstract in our last iteration of the manuscript but from your comment we deduce that you are not yet satisfied. We repeat your previous comments regarding the manuscript below for clarity.

*1. The abstract effectively identifies a methodological gap but provides little detail on how the two studies were structured or what types of robots and interaction settings were used. Clarifying the experimental design and the scope of interaction contexts would help readers better understand the validity and generalizability of the findings.

2. While the paper challenges the direct transfer of HHI measures to HRI, the abstract lacks a clear articulation of what new insights or frameworks emerge from this work. The authors should elaborate on how their findings advance the theoretical understanding of social mindfulness in HRI or guide future methodological development in robot interaction research.*

As stated by you we are very happy to hear we have responded your comments. We added information on how the studies were structured, the types of robots used and the experimental design, to allow for judgements regarding generalizability:

“Addressing these methodological shortcomings, the current studies investigated whether the SoMi task is a valid assessment of social mindfulness in HRI to determine under which conditions, if any, we can measure prosocial behavior towards robots. In Study One, participants interacted with a social robot (Cozmo) for three days, with perceived anthropomorphism and social mindfulness assessed before and after the interaction period. In Study Two, participants played the classic version of the SoMi paradigm using revised items matched in value for humans and robots, based on prior evaluations by a separate sample. Prolonged interaction with Cozmo did not increase social mindfulness but increased anthropomorphic perception of the robot. The revised items did not increase social mindfulness in the anthropomorphic condition, but they increased overall social mindfulness compared with previous studies.”

And we addressed validity and advances for theory and methodology in our abstract:

“Based on mixed findings, we stress that the field should carefully test HHI measures to ensure measurement validity before transferring them to HRI and that researchers must consider the context in which HRI occurs for external validity. Further, our findings contribute to theory on anthropomorphism as a dynamic process, raise new questions for theory on social mindfulness, and support the emerging critique of the widely used CASA paradigm.”

We believe that you were rightfully asking for the addition of these points and very much agree that they improved the abstract.

To react on your most recent comment we revised the abstract once more and tried to find a balance between what you asked us to add during the first review and the current comment.

1. *“lack more info from the results* “

We added some more information regarding the results and how they tie in with existing literature, future work and theory (additions marked in italics):

“Prolonged interaction with Cozmo did not increase social mindfulness but increased anthropomorphic perception of the robot. The revised items did not increase social mindfulness in the anthropomorphic condition, but they increased overall social mindfulness compared with previous studies. *We conclude that real-life interaction does not necessarily enhance social mindfulness towards robots, that the item selection and their value for both human and robots must be considered, and that future studies should explore other interaction time frames and items.* *Further, the increase in perceived anthropomorphism after a period of real-life interaction supports theory on anthropomorphism as a dynamic process.* More general, the results stress that the field should carefully test HHI measures to ensure measurement validity before transferring them to HRI and that researchers must consider the context in which HRI occurs for external validity.”

These are our main findings, and they answer 1) the question whether prolonged interaction with social robots increases social mindfulness and how it affects anthropomorphic perception of it, and 2) whether item value matters for the outcomes of the social mindfulness paradigm. This answers our main research questions. In case this was not the addition you were hoping for please point out specifically what you are missing.

2. *“it is overly lengthy on general information”*

While we are well within the limits set by PlosOne publishing guidelines the length of the abstract indeed increased. Hence, we tried to carefully shorten the abstract and remove redundant and overly general information. We say “carefully” as from your previous comment we understood that you missed some information on *“detail on how the two studies were structured or what types of robots and interaction settings were used” and experimental design and the scope of interaction contexts”* to enhance understandability of validity and generalizability for readers. We think you raised an excellent point there and wanted to keep this improvement. Nevertheless, we managed to cut the abstract from 399 to 341 words (see document for the full abstract). We hope that the current adjustments do justice on both your previous comments and your new comments.

*Reviewer #2: The authors have addressed all of my querries. I recommend the publication of this article to PLOS ONE Journal.*

Dear Reviewer 2,

Thank you for approving our manuscript. Through your comments were able to increase the quality of it and we are very thankful for that.

Kind regards,

The research team
